# Erythropoietin protects against rhabdomyolysis-induced acute kidney injury by modulating macrophage polarization

**DOI:** 10.1038/cddis.2017.104

**Published:** 2017-04-06

**Authors:** Shuo Wang, Chao Zhang, Jiawei Li, Sidikejiang Niyazi, Long Zheng, Ming Xu, Ruiming Rong, Cheng Yang, Tongyu Zhu

**Affiliations:** 1 Department of Urology, Zhongshan Hospital, Fudan University, Shanghai, China; 2 Shanghai Key Laboratory of Organ Transplantation, Shanghai, China; 3 Department of Transfusion, Zhongshan Hospital, Fudan University, Shanghai, China; 4 Shanghai Public Health Clinical Center, Fudan University, Shanghai, China

## Abstract

Erythropoietin (EPO) is a well-known hormone that is clinically used for the treatment of anemia. Very recently, an increasing body of evidence showed that EPO could still regulate bioactivities of macrophages. However, the details about the immunomodulatory effect of EPO on macrophages are not fully delineated, particularly in the setting of renal damages. Therefore, in the present study, we determined whether EPO could exert an impact on the dynamics of macrophages in a well-established model of rhabdomyolysis-induced acute kidney injury and explored the potential mechanisms. EPO was found to ameliorate kidney injuries by reducing macrophages recruitment and promoting phenotype switch toward M2 macrophages *in vivo*. It was also confirmed that EPO could directly suppress pro-inflammatory responses of M1 macrophages and promote M2 marker expression *in vitro*. Data indicated the possible involvement of Jak2/STAT3/STAT6 pathway in the augmentation of EPO on M2 polarization. These results improved the understanding of the immunoregulatory capacity of EPO on macrophages, which might optimize the therapeutic modalities of EPO.

Rhabdomyolysis, as the name indicates, is the disruption of striped (skeletal) muscle followed by massive leakage of electrolytes, myoglobin, and other intracellular proteins into the circulation.^1^ As a severer clinical condition, rhabdomyolysis can be caused by a broad range of physical or chemical damages such as trauma, physical exertion, infections, drugs and toxins, etc.^2^ Acute kidney injury (AKI) is a life-threatening complication of severe rhabdomyolysis and their association was first described during the World War II.^3^ Rhabdomyolysis is recognized now as one of the leading causes of AKI and rhabdomyolysis-induced AKI (RIAKI) accounts for ~10% of all AKI cases.^4, 5^ Current treatment for RIAKI relies on supportive therapies and the mortality remains considerably high.^6, 7, 8, 9^

A well-established animal model of rhabdomyolysis is induced by intramuscular injection of glycerol and widely used for the exploration of pathogenesis of RIAKI.^10^ The experimental data demonstrated that the mechanisms involved in the pathogenesis of RIAKI included direct injuries on tubular cells by iron release and oxidative stress from myoglobin, as well as indirect damages of subsequent renal vasoconstriction and tubular hypoxia.^11, 12, 13, 14, 15, 16^ Interestingly, recent studies shed light into the indispensable role of inflammation and immune cells during the process of RIAKI.^17, 18, 19, 20, 21, 22, 23^ Particularly, the dynamics and polarization of macrophages, to a great extent, contributed to the development and repair of renal damages.^19, 20, 21^ These studies suggest the macrophage as a novel target to develop effective therapeutics for RIAKI.

Erythropoietin (EPO) is a well-known hormone with classic hematopoietic functions and clinically used for the treatment of anemia. During the past decade, great interest has been paid to the pleiotropic biologic effects of EPO including anti-apoptosis, anti-inflammation, neurogenesis and angiogenesis, as well as their consequent tissue protection.^24, 25^ Recently, an increasing body of evidence showed that EPO could still modulate bioactivities of immune cells.^26, 27, 28, 29, 30^ Specifically for macrophages, EPO has been demonstrated to suppress pro-inflammatory cytokines and augment the phagocytic capacity.^26, 29, 30^ However, the details about the immunoregulatory effect of EPO remain elusive, particularly in the settings of various diseases. Therefore, in the present study, we explored whether EPO could exert an impact on the dynamics of macrophages in response to RIAKI and the potential mechanisms. Our results provided a novel recognition of EPO for macrophage regulation during AKI, which may help optimize the therapeutic strategies for EPO and develop effective EPO derivatives.

## Results

### EPO protected against rhabdomyolysis-induced kidney injury in mice

**Figure 1:**
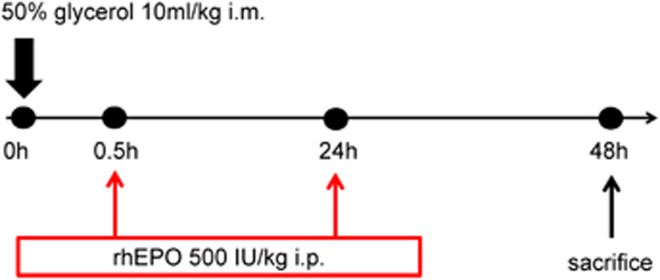
Experimental protocol for the induction of rhabdomyolysis-induced acute kidney injury in mice. 50% glycerol was administered intramuscularly into the hind limbs of mice at a dose of 10 ml/kg. Saline-injected mice were used as controls. To determine the protective effect of EPO, 500 IU/kg body weight of EPO or phosphate-buffered saline (PBS) was administered intraperitoneally at the indicated time point post-AKI. Forty eight hours after glycerol injection, mice were killed for blood and kidney samples

**Figure 2:**
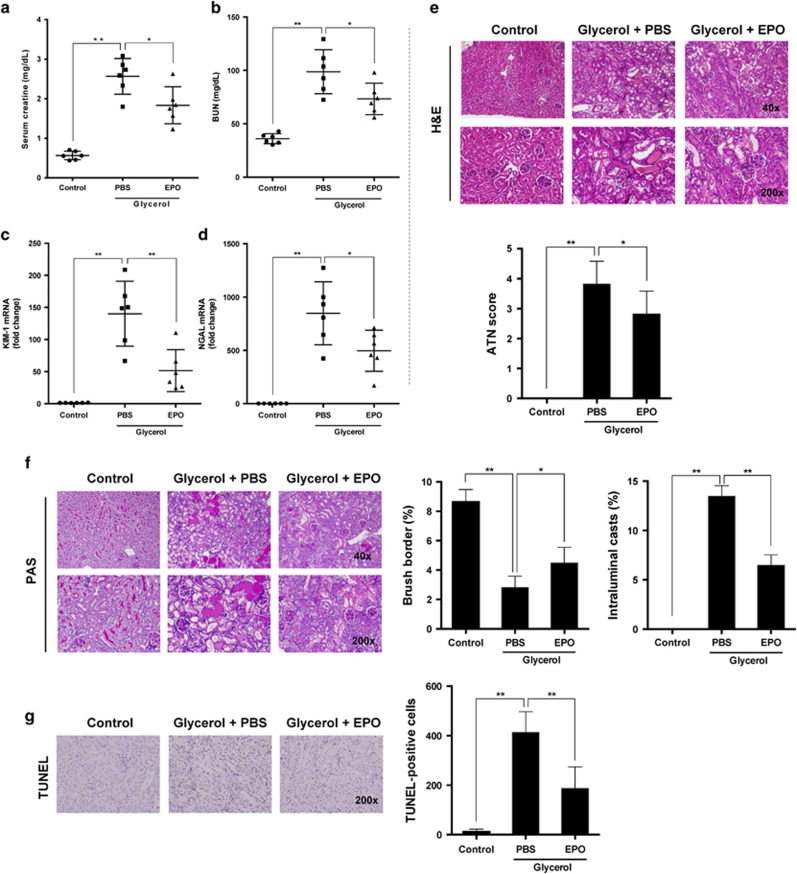
EPO improved renal damages in RIKAI. Mice subjected to different treatments were killed at the indicated time point. Serum levels of (**a**) creatinine and (**b**) blood urea nitrogen (BUN) were detected. Renal mRNA levels of (**c**) KIM-1 and (**d**) NGAL were analyzed by qPCR. (**e**) Representative images of H&E staining are exhibited and ATN scores were calculated. (**f**) Representative images of PAS staining and quantification of brush borders as well as intraluminal casts were showed. (**g**) Representative photographs of TUNEL staining (× 200). Quantitative analysis of TUNEL-positive cells was performed. Data are expressed as mean±S.D. (*n*=6). *P<0.05, **P<0.01

We initially confirmed the protection of EPO against rhabdomyolysis-associated renal injury in C57/BL6J mice. The protocols for rhabdomyolysis induction *in vivo* are summarized in Figure 1. EPO (rhEPO, 3SBIO, Inc., Shengyang, China) was administered at a dose of 500 IU/kg intraperitoneally 30 min and 24 h after glycerol (Sigma-Aldrich (Shanghai) Trading, Co., Ltd) injection. Glycerol injection caused obvious rises in serum creatinine and blood urea nitrogen (BUN) compared with saline-injected control mice. In contrast, treatment of EPO significantly improved kidney function (Figures 2a and b). In addition, renal mRNA expressions of kidney injury molecule-1 (KIM-1) and neutrophil gelatinase-associated lipocalin (NGAL) markedly increased after rhabdomyolysis induction while EPO administration significantly reduced KIM-1 as well as NGAL mRNA expression, consistent with renal function results (Figures 2c and d).

As shown in Figure 2e, intramuscular injection with 50% glycerol resulted in severe damage of kidney structures especially at the corticomedullary junction, characterized by tubular necrosis, tubular dilation, and cast formation. ATN score was used to grade the histologic alterations based on hematoxylin and eosin (H&E) staining. EPO obviously improved tubular injury and lowered the ATN score (Figure 2e) in contrast to those of phosphate-buffered saline (PBS)-treated mice. More precisely, we performed periodic acid-Schiff (PAS) staining to clearly exhibit intratubular cast and brush border loss. Although both intratubular cast and brush border stain strongly with PAS, their structures can be easily distinguished due to the paucity of nuclei for intratubular casts (Figure 2f). The extent of casts and preservation of brush borders were further semiquantitatively analyzed. In accordance to the morphologic changes in H&E-stained slides, treatment of EPO preserved more brush borders and diminished intraluminal casts as shown in Figure 2f.

To evaluate apoptosis in kidney tissues *in situ* during RIAKI, TdT mediated dUTP nick end labeling (TUNEL) staining was conducted. Subjection to glycerol caused a significant rise in apoptotic cells at the corticomedullary junction. Consistent with kidney function and morphology, administration of EPO suppressed apoptosis markedly (Figure 2g).

### EPO ameliorated RIAKI-associated renal inflammation

**Figure 3:**
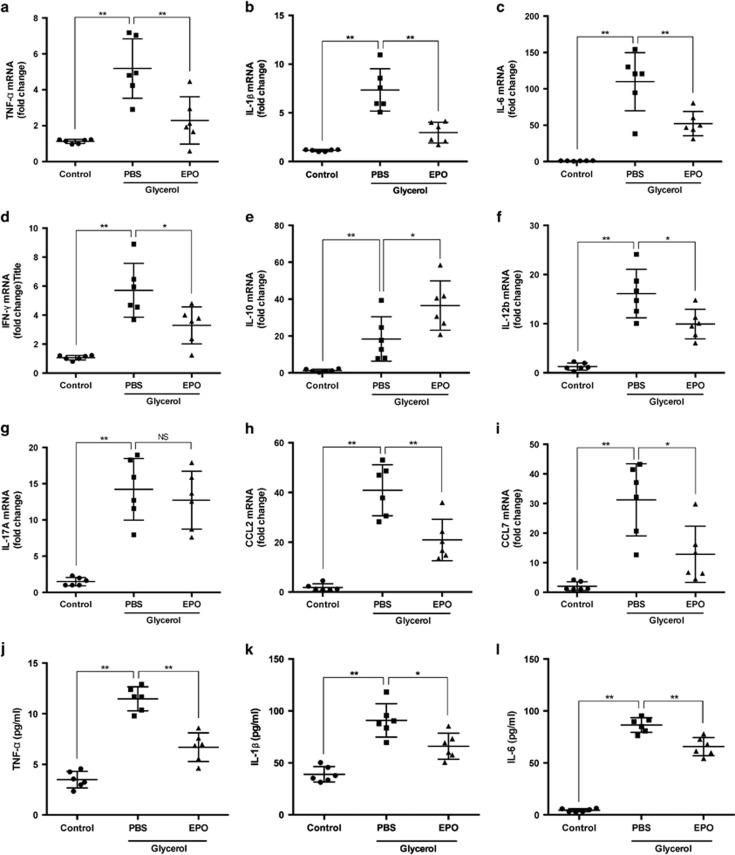
EPO alleviated inflammatory responses in RIAKI. Renal mRNA levels of (**a**) TNF-*α*, (**b**) IL-1*β*, (**c**) IL-6, (**d**) IFN-*γ*, (**e**) IL-10, (**f**) IL-12b, (**g**) IL-17 A, (**h**) CCL2, and (**i**) CCL7 were analyzed by qPCR. Serum levels of (**j**) TNF-*α*, (**k**) IL-1*β*, and (**l**) IL-6 were determined. Data are expressed as mean±SD (*n*=6). *P<0.05, **P<0.01

Renal mRNA expressions of cytokines and chemokines were detected by real-time quantitative PCR (RT–qPCR) to evaluate the inflammatory state of kidneys. Levels of pro-inflammatory cytokines including TNF-*α*, IL-1*β*, IL-6, IL-12b, IFN-*γ* and chemokines including C-C motif chemokine ligand 2 (CCL2) and 7 (CCL7) significantly increased after rhabdomyolysis induction compared with PBS-treated mice, and these upregulations were partially reversed by treatment of EPO (Figures 3a–f). Similar results were also obtained in the determination of TNF-*α*, IL-1*β* and IL-6 levels in murine serum (Figures 3j–l). These results were consistent with functional and histologic data and indicated an immunomodulatory role of EPO.

### EPO modulated macrophage infiltration and polarization in kidneys during RIAKI

**Figure 4:**
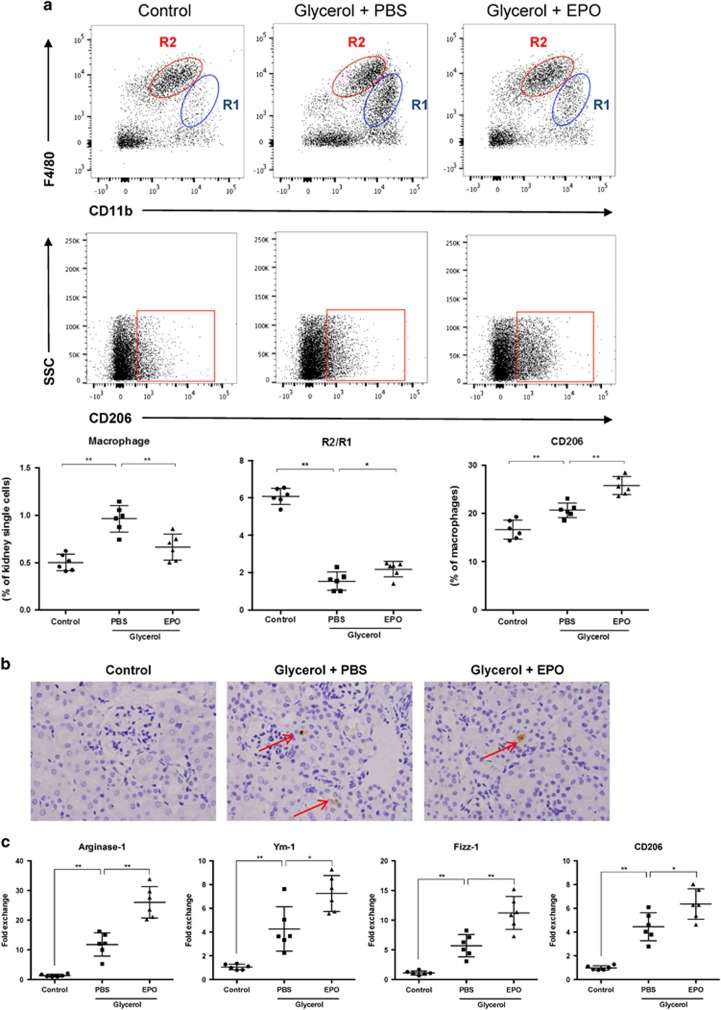
EPO diminished macrophage infiltration and regulated macrophage polarization within kidneys during RIAKI. (**a**) Intact kidneys were decapsulated, diced, and digested for single cell suspensions. Renal single cells were then incubated with anti-mouse CD45, CD11b, F4/80 and CD206 antibodies and subsequently analyzed by flow cytometry. Macrophages can be divided into two subgroups: CD11b^high^F4/80^low^ (R1), and CD11b^+^F4/80^high^ (R2). Quantitative analysis of macrophages (CD11b^+^F4/80^+^) percentage, ration of R2/R1, and M2 (CD206^+^) percentage was performed. (**b**) Representative photographs of anti-F4/80 staining at × 200 magnification. Arrows suggested macrophages located within renal interstitium. (**c**) Renal mRNA levels of arginase-1, Ym-1, Fizz-1 and CD206 were detected by qPCR. Data are expressed as mean±S.D. (*n*=6). *P<0.05, **P<0.01

Considering macrophage plays a crucial role in the development of RIAKI, we further explored the infiltration and phenotypes of macrophages. By flow cytometry, pan-leukocyte marker CD45 and macrophage marker CD11b, F4/80 were used to distinguish macrophages in kidney single cell suspension. Gating strategy was exemplified in Supplementary Figure S1. Cells with both expressions of CD11b and F4/80 were recognized as macrophages and two populations could be distinguished: CD11b^high^F4/80^low^ (R1), and CD11b^+^F4/80^high^ (R2). The percentage of CD11b^+^F4/80^+^ cells among whole renal cells was calculated and served as a parameter for the infiltration of macrophages. As shown in Figure 4a, mice subjected to glycerol demonstrated a dramatic recruitment of macrophages toward kidneys while treatment of EPO alleviated such infiltration significantly. The dynamics of subpopulations R1 and R2 was also analyzed. R2 cells are recognized as the resident macrophages within kidneys and presents with M2-like phenotype. Accordingly, our results confirmed the predominance of R2 in kidneys from control group, and demonstrated a dramatic increase in R1 population after rhabdomyolysis induction. However, the ratio of R2/R1 at 2 days after glycerol injection was partial reversed by EPO administration. More accurately, anti-CD206 antibody was used to determine the M2 phenotype. The data showed that the proportion of M2 macrophages increased during RIAKI and was further upregulated by EPO. Additionally, immunohistochemical staining for F4/80 was performed and confirmed the flow cytometry data that macrophages infiltration was diminished by EPO (Figure 4b). Importantly, mRNA levels of M2 markers including arginase-1, Ym-1, Fizz-1 and CD206 were examined by RT–qPCR and suggested EPO could enhance polarization toward M2 macrophages (Figure 4c).

### EPO inhibited apoptosis and reduced chemokine production of tubular epithelial cells in response to myoglobin

**Figure 5:**
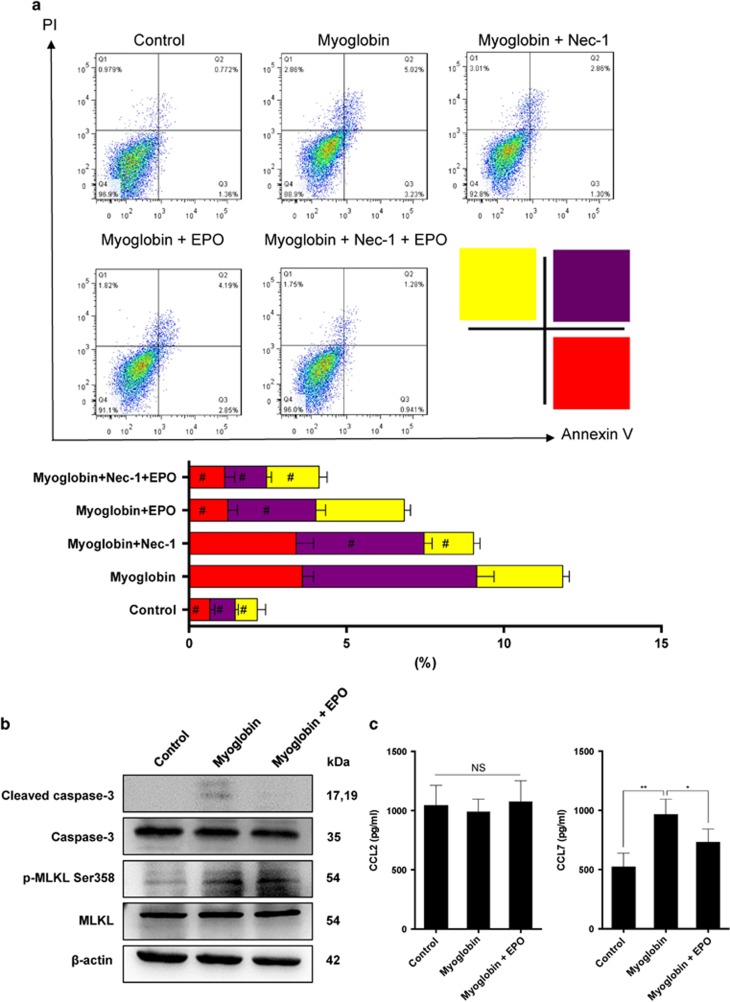
EPO inhibited myoglobin-induced apoptosis and chemokine production of tubular epithelial cells *in vitro*. Human renal proximal tubular epithelial cell line HK-2 was treated with 10 mg/ml myoglobin for 4 h in the presence or absence of 50 IU/ml EPO. (**a**) To explore a role of necroptosis in myoglobin-induced cell death, a necroptosis inhibitor Nec-1 (10 *μ*M) was used. Cells were collected and detected by flow cytometry based on Annexin V/PI staining. (**b**) Protein was prepared from cultured cells. Expressions of cleaved caspase-3 and phosphorylated MLKL were analyzed by western blotting. (**c**) Levels of CCL2 and CCL7 in the culture supernatants were determined by ELISA. Data are representative of three independent experiments. Data are expressed as mean±S.D. *P<0.05, **P<0.01. ^#^*P*<0.05 compared with myoglobin-treated groups

Cell death serves as an important initiator of recruitment of immune cells and subsequent inflammatory responses. The anti-apoptotic effect of EPO has been widely reported and was recognized as a key contributor to its renoprotection. However, the current prevailing theory believes that necroptosis, a novel identified cell death, plays a crucial role in the pathogenesis of AKI, even outweighs apoptosis.^31, 32, 33^ Therefore, it is indeed necessary to explore whether EPO could modulate necroptosis in the setting of RIAKI. Therefore, we subjected human renal proximal tubular epithelial cell line (HK-2) to myoglobin to mimic rhabdomyolysis-associated kidney injuries *in vitro*. Graded concentrations of myoglobin (1, 5, and 10 mg/ml) were tested, and only at 10 mg/ml could myoglobin induce obvious cell death (data not shown). Flow cytometry analysis was conducted to detect different types of cell death based on Annexin V/PI staining: Annexin V^−^/PI^−^ are live cells; Annexin V^+^/PI^−^ represent early apoptosis; Annexin V^−^/PI^+^ reflect necroptosis; Annexin V^+^/PI^+^ include both late apoptosis and necroptosis. In accordance with previous studies,^21, 34^ exposure to myoglobin caused a significant decrease in cell viability in contrast with vehicle-treated HK-2 cells. Moreover, addition of Nec-1, a widely used inhibitor for necroptosis, obviously diminished PI^+^ cell death, indicating myoglobin could induce necroptosis as well. The results also demonstrated that treatment of EPO diminished Annexin V^+^ cell death (Figure 5a). Considering Annexin V^+^/PI^+^ contained an overlapping cell spectrums of both necroptosis and apoptosis, western blot was further used to delineate the effects of EPO on necroptosis and apoptosis separately. Activated hRIP3 directly phosphorylates hMLKL at the Thr357 and Ser358 sites, phosphorylated hMLKL has been recognized as useful biomarkers for the characterization of necroptosis *in vitro.*^35^ As shown in Figure 5b, EPO decreased the level of cleaved caspase-3 but had no impact on phosphorylated MLKL (Ser358), suggesting EPO could only inhibit apoptosis but not necroptosis that is more pro-inflammatory. Considering CCL2 and CCL7 play key roles in the infiltration of macrophages,^36^ we further determined the secretion of CCL2 and CCL7 in HK-2 cells in response to myoglobin. Myoglobin caused an increase in the production of CCL7 and treatment of EPO partially reversed the CCL7 level (Figure 5c). Overall, these data suggested that EPO could suppress the recruitment of macrophages by inhibiting apoptosis and chemokine secretion.

### EPO facilitated macrophage polarization toward alternative activation (M2) *in vitro*

**Figure 6:**
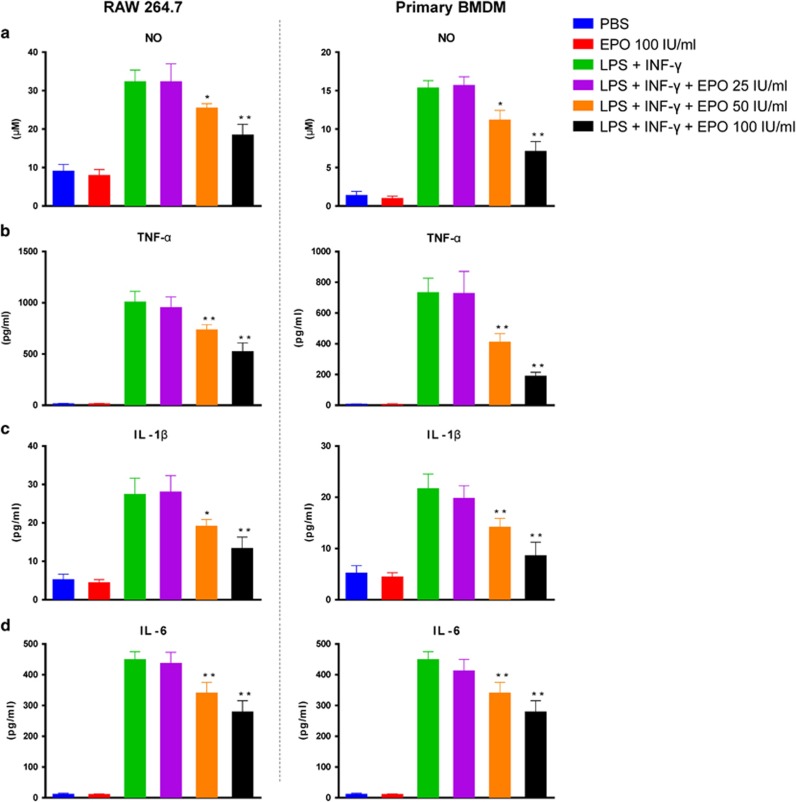
EPO inhibited pro-inflammatory response in M1 macrophages. RAW 264.7 cells and bone marrow-derived macrophages (BMDM) were primed with 1 *μ*g/ml LPS and 20 ng/ml IFN-*γ* for 24 h. EPO were added at indicated concentrations. Secretion levels of (**a**) NO, (**b**) TNF-*α*, (**c**) IL-1*β*, and (**d**) IL-6 were examined by Griess reaction and ELISA. Experiments were performed in triplicate. Data are expressed as mean±S.D. *P<0.05, **P<0.01

**Figure 7:**
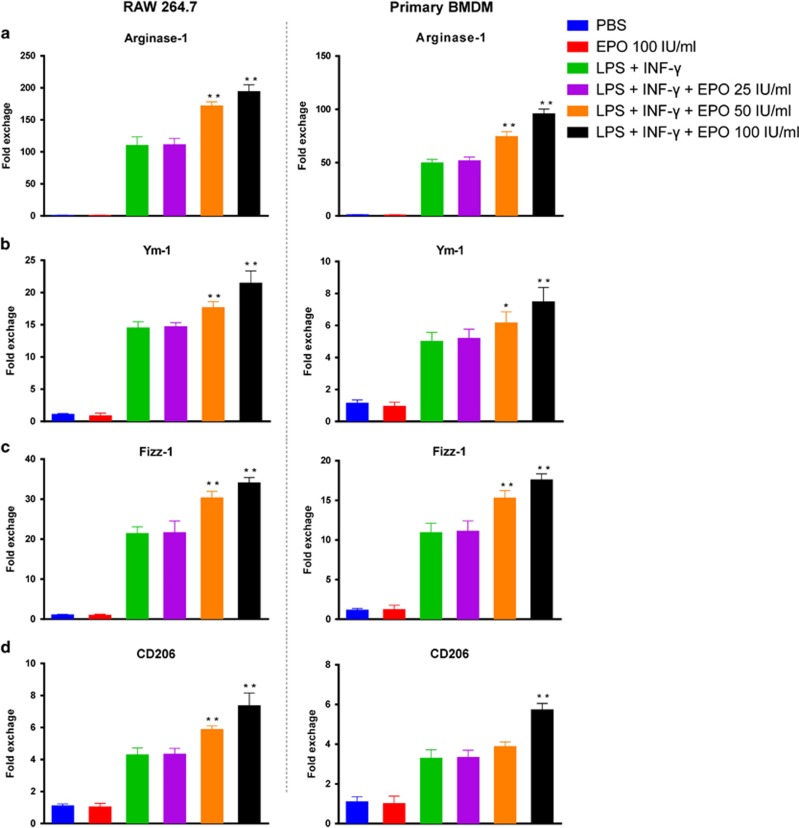
EPO promoted the expression of M2 markers. RAW 264.7 cells and bone marrow-derived macrophages (BMDM) were stimulated with 20 ng/ml IL-4 for 24 h. EPO were added at indicated concentrations. Cellular mRNA expressions of (**a**) arginase-1, (**b**) Ym-1, (**c**) Fizz-1, and (**d**) CD206 were detected by qPCR. Experiments were performed in triplicate. Data are expressed as mean±S.D. *P<0.05, **P<0.01

Next, we determined whether EPO was able to regulate macrophage phenotypes directly. As shown in Figure 6, EPO reduced the concentrations of pro-inflammatory molecules including NO, TNF-*α*, IL-1*β*, and IL-6 in a dose-dependent manner in both RAW264.7 macrophage cell line and primary bone marrow-derived macrophage (BMDM), which were stimulated by a combination of LPS and IFN-*γ*. Alternative activation of macrophages (M2) was initiated by addition of IL-4. Importantly, co-treatment of IL-4 and EPO upregulated the mRNA expressions of arginase-1, Ym-1, Fizz-1, and CD206 which were indicators of M2 macrophages (Figure 7). Notably, only in the presence of IL-4 can EPO promote M2 polarization in macrophages, while EPO alone exerted no effect. Taken together, those results indicated that EPO inhibited M1 and augmented M2 phenotypes, and thereby favored an immunoregulatory switching of macrophages.

### Jak2/STAT3/STAT6 pathway was involved in the promotion of M2 phenotype by EPO

**Figure 8:**
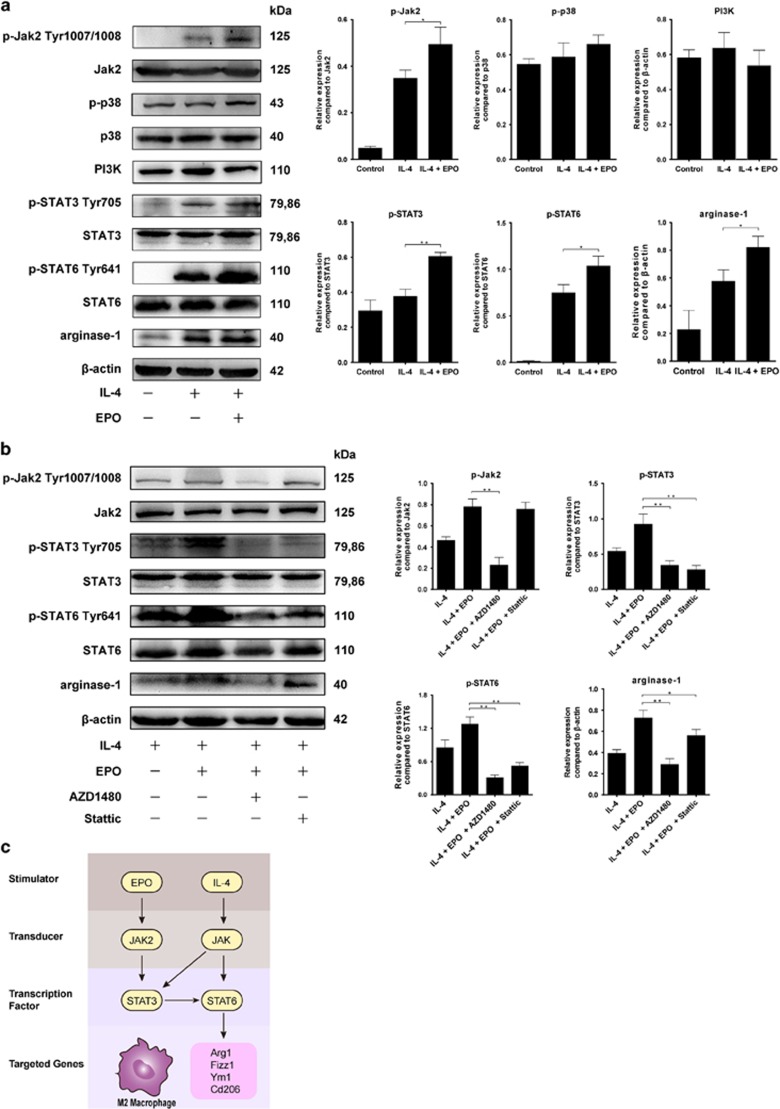
EPO augmented M2 polarization via Jak2/STAT3/STAT6 pathway. (**a**) RAW 264.7 cells were subjected to 20 ng/ml IL-4 in the presence or absence of EPO (50 IU/ml) for 24 h. Protein expressions of related molecules were measured and representative photographs are exhibited. (**b**) To further confirm the contribution of Jak2/STAT3, RAW 264.7 cells stimulated by IL-4 and EPO were incubated with AZD1480 (an inhibitor of p-Jak2, 5 *μ*M) or Stattic (an inhibitor of p-STAT3, 10 *μ*M). Protein expressions of related molecules were measured and representative images are demonstrated. (**c**) Proposed pathways by which EPO promotes M2 polarization in the presence of IL-4. Experiments were performed in triplicate. Error bars represent S.D. *P<0.05, **P<0.01

We subsequently explored the possible mechanism by which EPO promotes M2 polarization. It is well documented that EPO activates a series of signaling pathways including Janus-tyrosine kinase 2 (Jak2)/signal transducer and activator of transcription (STAT), phosphoinositide 3 kinase (PI3K), mitogen-activated protein kinase (MAPK) pathways. In this regard, we evaluated the key participants of these pathways by western blot and found significant upregulations of p-Jak2, p-STAT3, and arginase-1 in EPO-treated macrophages (Figure 8a). Alterations in PI3K and p-p38 expression were also observed but did not reached the statistical significance. Considering EPO could not promote M2 polarization alone, we also determined the expression of p-STAT6, which is a key regulator in the generation of IL-4-stimulated M2 macrophage. We further determined the contribution of Jak2/STAT3 to EPO-induced M2 polarization by applying p-Jak2 inhibitor AZD1480 (Selleck) and p-STAT3 inhibitor Stattic (Selleck). As shown in Figure 8b, both inhibitors abolished the EPO-mediated increases in p-STAT6 and arginase-1, indicating that EPO might enhance M2 polarization by upregulating p-STAT6 via Jak2/STAT3 pathway. Proposed pathways was illustrated in Figure 8c.

## Discussion

**Figure 9:**
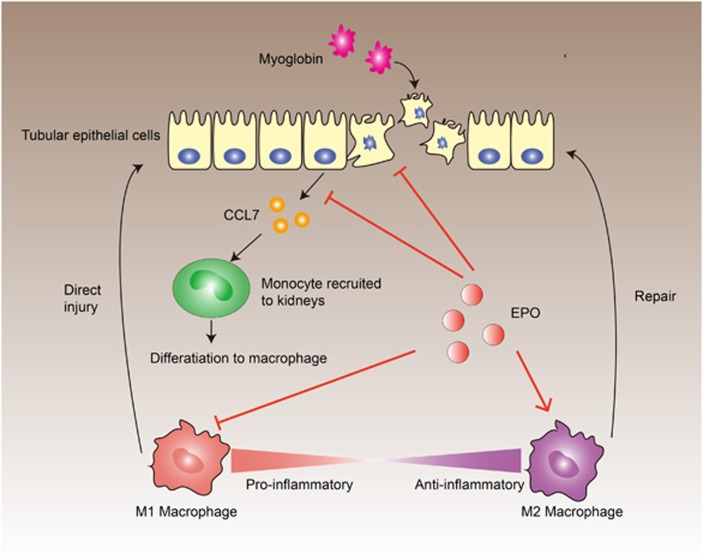
Proposed mechanisms involved in the modulation of macrophage phenotypes by EPO during rhabdomyolysis-induced acute kidney injury. Myoglobin can induce both necroptosis and apoptosis of tubular epithelial cells and initiate the secretion of chemokine CCL7, which recruit monocytes and serve as key initiator of the subsequent inflammation. EPO could only inhibit apoptosis, but not necroptosis that are more destructive and pro-inflammatory. On the other hand, EPO decreases the level of secreted chemokine CCL7, thus consequently suppresses the migration of monocytes into kidneys. Moreover, EPO can directly inhibit the activation of M1 subtypes and promotes polarization toward M2 phenotypes. As a result, the M1/M2 ratio during rhabdomyolysis-induced acute kidney injury is favorably regulated toward an anti-inflammatory phenotype and thereby ameliorates the renal damages

Despite of an increased insight into the anti-inflammatory effect of EPO in the past decade, the modulation of EPO on immune cells and their exact mechanisms are not fully elucidated. A better understanding of the immunoregulatory capacity of EPO may improve the therapeutic regimens of EPO in the treatment for various diseases. In the present study, we demonstrated that EPO could diminish the rhabdomyolysis-associated kidney injuries by ameliorating the infiltration of macrophages and promoting M2 polarization within kidneys during RIAKI. As shown in Figure 9, EPO exerted its modulation on the dynamics of renal macrophages through pleiotropic mechanisms. Myoglobin caused direct injuries on the tubular epithelial cells and stimulated secretion of chemokine CCL7. In contrast, EPO protected against myoglobin-induced apoptosis and decreased the level of CCL7, thereby inhibiting the recruitment of monocytes/macrophages. In addition, EPO suppressed the pro-inflammatory responses of activated macrophages. More importantly, our data showed that EPO were able to augment the polarization of macrophages toward M2 phenotype in the presence of IL-4. By these mechanisms, EPO significantly altered the paradigm of macrophages in the microenvironment of kidneys and thereby reduced M1-related tissue damages and enhanced the reparative effects of M2. Our research provided a novel recognition of EPO on macrophage subtype switch specifically in the setting of AKI.

An increasing body of studies has shed light into the effects of EPO on macrophage bioactivities. Several studies have proved that EPO could inhibit pro-inflammatory activation of macrophages.^26, 37, 38^ In accordance with our results, EPO has also been shown to promote macrophage-mediated T cell suppression^39^ and induce the expansion of M2-like macrophages in white adipose tissue.^40^ These characteristics have also been confirmed in nonerythropoietic EPO derivative ARA290.^41, 42, 43, 44^ However, another study obtained the conflicting results that EPO enhanced the pro-inflammatory activity of macrophages.^45^ It remains unknown what results in this discrepancy, which needs further explorations in the future. Interestingly, EPO has been demonstrated to enhance the phagocytic function of macrophages.^29, 30, 37^ Clearance of damaged tissue can facilitate renal repair and promote immune tolerance. Therefore, the augmentation of phagocytosis of macrophages is likely to be one of the mechanisms for the renoprotective effect of EPO as well. Such hypothesis does not contradict with our study, in which we focused the functional switch of macrophages.

Commonly defined by *in vitro* experiments, macrophage activation can be distinguished for two major subtypes: classically activated M1 macrophages are pro-inflammatory and exert deleterious effect in sterile tissue injuries; in contrast, alternatively activated M2 macrophages are characterized by immunoregulatory and tissue repair capabilities.^46, 47, 48^ Although indeed simplistic, this essential classification is well accepted and explains the functional plasticity and dynamics of macrophages in response to different insults *in vivo.*^49^ Typically, renal macrophages are a heterogeneous group with their phenotypes changing under physiological and pathological conditions.^50^ Normally, there are a small number of resident macrophages within kidneys that are of vital importance for maintaining homeostasis of renal microenvironments.^51, 52^ Upon outsets of diverse AKI, primary renal damages are inevitably followed by a rapid influx of abundance of monocytes that subsequently differentiate into macrophages.^53, 54^ In the early stage of AKI, infiltrated macrophages present as pro-inflammatory M1 phenotype and consequently amplify tissue damage by secretion of cytokines and induction of apoptosis. Over a period of time, the predominance of macrophages switches to the anti-inflammatory M2 subtype, which facilitate post-AKI repair.

Endogenous factors in the kidney microenvironment regulate the polarization dynamics of macrophages and the underlying mechanisms are under intensively investigation recently. Retinoic acid, colony-stimulating factors 1 and 2 have been demonstrated to promote the alternative activation of macrophages during AKI.^55, 56, 57, 58, 59^ The dynamic equilibrium between M1 and M2 macrophages plays a crucial role in the initiation and development of kidney damages. In this regard, it is of great value to explore how to therapeutically manipulate this balance favorably toward M2 polarization. Mesenchymal stem cells, for instance, have been reported to improve RIAKI by activation of M2 macrophages.^20^

Previous research observed that adoptive transfer of M2 macrophages led to an attenuation of renal pathology.^60^ In this study, we showed that EPO could facilitate M2 polarization via Jak2/STAT3/STAT6 pathway that may suggest another mechanism of the tissue protection of EPO against AKI. Of note, the phenotypic evolving of renal macrophages during AKI – as discussed above – is a complex process, which is orchestrated by diverse factors. In our study, however, we simplified M2 polarization *in vitro* by using classic IL-4-primed macrophages. Strictly, macrophage-specific *epo receptor* knockout mice model are needed to further identify the effect of EPO on macrophages *in vivo* and *in vitro*, which is warranted in the future study.

Moreover, a number of reports indicated that M2 macrophages within kidneys could promote renal fibrosis in addition to their reparative effects on tissue damages.^61, 62^ In this regard, although there is no solid evidence suggesting the side effect of EPO on fibrosis in the treatment for AKI, the possible risk should take into account in the following researches.

In conclusion, the present study demonstrated that EPO could reduce macrophages recruitment and promote phenotype switch toward M2 macrophages during RIAKI. We also proved that EPO directly suppressed pro-inflammatory responses of M1 macrophages and promoted M2 marker expression *in vitro*. These results improve the understanding of the immunomodulatory capacity of EPO on macrophages, especially in the setting of AKI, which might optimize the therapeutic modalities of EPO for sterile kidney injuries. A deep insight into the mechanisms of the immunomodulation of EPO can also help develop novel EPO derivatives with immunoregulatory functions.

## Materials and methods

### Animals and rhabdomyolysis-induced AKI model

The Ethics Committee of Zhongshan hospital, Fudan University approved this study. All animal experiments were performed in accordance with established guidelines for the care and use of laboratory animals. C57/BL6J mice (male, 6–8 weeks old) were purchased from SLAC Laboratory (Shanghai, China), and housed in a pathogen-free, temperature-controlled environment with a 12-h light/dark cycle. Animals had free access to food and water. To induce rhabdomyolysis, mice were intramuscularly injected with 50% glycerol (Sigma-Aldrich, Shanghai, China, 10 ml/kg) into the hind limbs or saline as a control. To explore the possible protection of EPO against RIAKI, 500 IU/kg body weight of recombinant human EPO or PBS was administered intraperitoneally 30 min and 24 h after the glycerol injection. On day 2 after glycerol treatment, blood and kidney samples were collected for various detections.

### Detection of kidney function

Serum creatinine and BUN, serving as indicators of kidney function, were measured using QuantiChrom Creatinine Assay Kit and QuantiChrom Urea Assay Kit (BioAssay Systems, Hayward, CA, USA) separately based on colorimetric assay according to the manufacturer's protocol.

### Kidney histology

For histology, kidneys were fixed in 4% paraformaldehyde for 24 h and embedded in paraffin. Paraffin-embedded kidney blocks were cut into 2-*μ*m sections and then subjected to routine hematoxylin and eosin (H&E) staining as well as periodic acid-Schiff (PAS) staining. Tissue sections were viewed by light microscope at × 200 magnification. For semiquantitative analysis, five different fields at corticomedullary junction from each group were randomly selected. The histologic alterations in H&E-stained slides were indicated as ATN score, which is graded on a scale of 0–5 according to tubular necrosis, tubular dilation and cast formation (0, none; 1, <11% 2, 11% to 25% 3, 26% to 45% 4, 46% to 75% 5, >75%). The percentage area of PAS-stained brush border as well as tubular casts was quantified by using ImageJ software. Such histologic evaluations were performed in a blinded manner by experienced researchers.

### TdT mediated dUTP nick end labeling (TUNEL) assay

To detect renal apoptosis in response to RIAKI, TUNEL staining was performed using *In Situ* Cell Death Detection Kit (Roche, Indianapolis, IN, USA) according to the manufacturer's instruction. The number of apoptotic cells was counted under light microscope at × 200 magnification. At least 5 areas at the corticomedullary junction in the sections from different mice of each group were determined and averaged.

### Flow cytometry

Intact kidney tissues were decapsulated, diced, and digested in collagenase type IV (1 mg/ml, Stemcell Technologies, Vancouver, British Columbia, Canada) for 30 min at 37 °C. Kidney tissue suspension was then passed through 40 *μ*m Falcon meshes followed by red cell lysis. Single cell suspension was washed 3 times with PBS and incubated with anti-mouse CD45-Brilliant Violet 510, CD11b-PE/Cy7, F4/80-Alexa Fluor 647 and CD206-PE (Biolegend, SanDiego, CA, USA) for 1 h at 4 °C and detected on FACSCaliber Analyzer (BD Biosciences, San Jose, CA, USA). Data was processed using FlowJo software (Ashland, OR, USA).

### Immunohistochemistry

For immunohistochemical staining of F4/80, paraffin-embedded kidney sections were rehydrated through a series of graded ethanol and then incubated in 0.3% hydrogen peroxide to block the endogenous peroxidase activity. Specific primary antibodies were used for the detection of F4/80 (1:100, Abcam, Cambridge, MA, USA).

### Cell culture and treatment

Human renal proximal tubular epithelial cell line (HK-2) was obtained from ATCC and maintained in DMEM/F12 medium containing 10% FBS. HK-2 cells were treated with 10 mg/ml myoglobin to mimic rhabdomyolysis *in vitro*. Different concentrations (25, 50, and 100 IU/ml) of EPO and Nec-1 (10 *μ*M) were also used. HK-2 cells maintained in normal medium were used as the control.

Macrophage cell line RAW 264.7 was purchased from ATCC and maintained in DMEM medium containing 10% FBS. Bone marrow cells from mice tibia and femur were incubated with 1000 U/ml GM-CSF (PeproTech) for 5 days to generate bone marrow-derived macrophages (BMDM). LPS (1 mg/ml, Sigma-Aldrich)+IFN-*γ* (20 ng/ml, PeproTech, Southfield, MI, USA) or IL-4 (20 ng/ml, PeproTech) were used for M1 or M2 polarization in RAW 264.7 cells and BMDM, respectively.

### Annexin V/PI assay

Cell death was analyzed using Annexin V-FITC/PI apoptosis detection kit (Vazyme Biotech, Nanjing, China). According to the manufacturer's protocol, cells were collected and resuspended in 100 *μ*l binding buffer mixed with 5 *μ*l Annexin V-FITC reagent and 5ul PI reagent. After incubation for 15 min at room temperature in the dark, another 400 *μ*l binding buffer was added, and cells were measured by flow cytometry (Beckman Coulter, Fullerton, CA, USA). Data was analyzed with FlowJo software. Annexin V^−^/PI^−^ represents live cells; Annexin V^+^/PI^−^ represents early apoptotic cells; Annexin V^−^/PI^+^ reflects necrosis; double positivity included both late apoptosis and necrosis.

### ELISA and Griess reaction

Murine TNF-*α*, IL-1*β*, IL-6, and human CCL2, CCL7 ELISA kit were purchased from R&D Systems (Boston, MA, USA). The levels of these cytokines and chemokines in serum or culture supernatants were measured according to the manufacturer's protocols. NO level in culture supernatants was detected by griess reagent (Sigma-Aldrich) according to the manufacturer's instruction.

### Real-time quantitative PCR

Total RNA was extracted from kidney tissues and cultured cells using Trizol reagent according to the manufacturer's protocol. cDNA was reverse-transcribed using Reverse Transcription Kit (Takara, Dalian, China). RT–qPCR was performed using SYBR Green PCR mix (Roche) on an ABI Prism 7500HT Sequence Detection System (Applied Biosystems, Paisley, UK). Gene expression levels were presented as fold exchange that was normalized to *β*-actin in control group. Primer sequences are listed in Table 1.

### Western blotting

Total proteins from cultured cells were separated on SDS-polyacrylamide gels and transferred onto nitrocellulose membranes. The membranes were blocked in 5% nonfat milk for 1 h at room temperature, and then incubated overnight at 4 °C with primary antibodies against cleaved caspase-3, caspase-3, p-Jak2 tyr1007/1008, Jak2, p-p38, p38, PI3K, p-STAT3, STAT3, p-STAT6, STAT6, arginase-1, *β*-actin (Cell Signaling Technology, 1:1000), MLKL and p-MLKL ser358 (Abcam, 1:1000). Blots were subsequently washed and incubated with secondary antibodies for 1 h at room temperature. The immunoblots were visualized by chemiluminescence and quantified using ImageJ software.

### Statistical analysis

All data are presented as means±S.D. Statistical analysis was performed using the Student's *t*-test (between two groups) and one-way ANOVA (among three or more groups) by SPSS 19.0 software (SPSS, Inc., Armonk, NY, USA). The Scheffe test was used for *post hoc* analysis. *P*<0.05 was recognized as statistically significant.

## Figures and Tables

**Table 1:** Primers used for qPCR analysis

**Genes**	**Forward primer (**5′-3′**)**	**Reverse primer (**5′-3′**)**
*KIM-1*	TCCACACATGTACCAACATCAA	GTCACAGTGCCATTCCAGTC
*NGAL*	CCATCTATGAGCTACAAGAGAACAAT	TCTGATCCAGTAGCGACAGC
*TNF-α*	TCTTCTCATTCCTGCTTGTGG	GGTCTGGGCCATAGAACTGA
*IL-1β*	TGTAATGAAAGACGGCACACC	TCTTCTTTGGGTATTGCTTGG
*IL-6*	GATGGATGCTACCAAACTGGA	CCAGGTAGCTATGGTACTCCAGAA
*IL-10*	ACCTGGTAGAAGTGATGCCCCAGGCA	CTATGCAGTTGATGAAGATGTCAAA
*IL-12b*	AGGTCACACTGGACCAAAGG	AGGGTACTCCCAGCTGACCT
*IL-17 A*	TCCAGAAGGCCCTCAGACTA	CTCGACCCTGAAAGTGAAGG
*IFN-γ*	GAACTGGCAAAAGGATGGTGA	TGTGGGTTGTTGACCTCAAAC
*CCL2*	GTCCCTGTCATGCTTCTGG	GCTCTCCAGCCTACTCATTG
*CCL7*	TCTCTCACTCTCTTTCTCCACC	GGGATCTTTTGTTTCTTGACATAGC
*Arginase-1*	GTGAAGAACCCACGGTCTGT	CTGGTTGTCAGGGGAGTGTT
*Ym-1*	TTCTTGTCACAGGTCTGG	TCCTTAGCCCAACTGGTATAG
*Fizz-1*	AGGAACTTCTTGCCAATCCA	CTGGGTTCTCCACCTCTTCA
*CD206*	CCTGTGCTCGAGAGGATATG	GCAGTCTGCATACCACTTGT
*β-actin*	CCACGAGCGGTTCCGATG	GCCACAGGATTCCATACCCA
