# Supplementary figures and images for: Erythropoietin protects against rhabdomyolysis-induced acute kidney injury by modulating macrophage polarization
Source: Cell Death Dis. 2017 Apr 6;8(4):e2725–. doi: 10.1038/cddis.2017.104 (PMC5477572; doi:10.1038/cddis.2017.104)

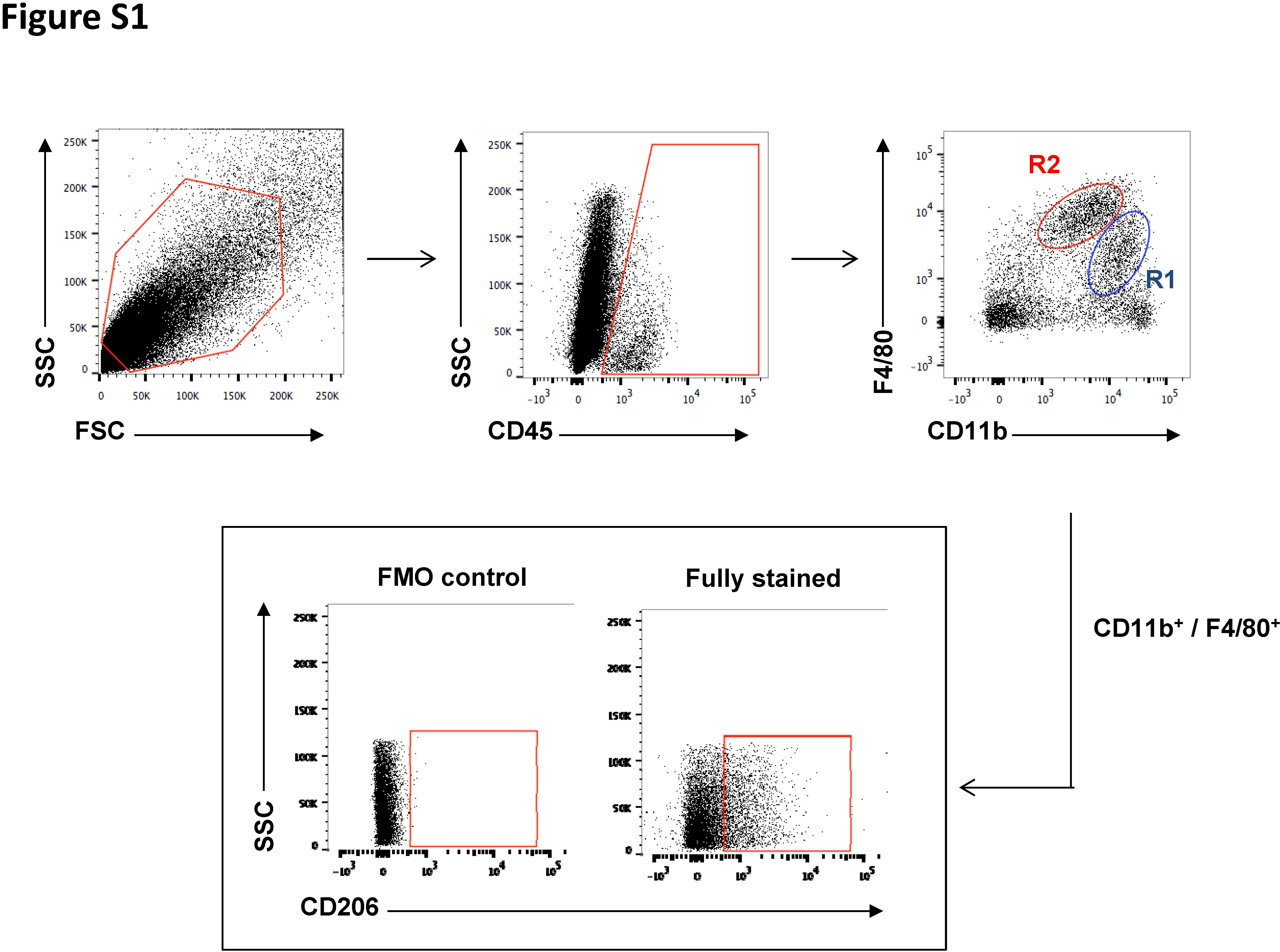

Supplement: Supplementary Figure [file cddis2017104x1.tif]
